# Scalable approaches for generating, validating and incorporating data from high-throughput functional assays to improve clinical variant classification

**DOI:** 10.1007/s00439-024-02691-0

**Published:** 2024-08-01

**Authors:** Samskruthi Reddy Padigepati, David A. Stafford, Christopher A. Tan, Melanie R. Silvis, Kirsty Jamieson, Andrew Keyser, Paola Alejandra Correa Nunez, John M. Nicoludis, Toby Manders, Laure Fresard, Yuya Kobayashi, Carlos L. Araya, Swaroop Aradhya, Britt Johnson, Keith Nykamp, Jason A. Reuter

**Affiliations:** 1 Invitae Corporation, San Francisco, CA 94103 USA; 2 Epic Bio, South San Francisco, CA 94080 USA; 3 Present Address: Calico Life Sciences, South San Francisco, CA 94080 USA; 4 Present Address: Gilead Life Sciences Inc, Foster City, CA 94404 USA; 5 Present Address: Department of Structural Biology, Genentech, South San Francisco, CA 94080 USA; 6 Present Address: GeneDx, Stamford, CT 06902 USA; 7 Present Address: Tapanti.org, Santa Barbara, CA 93108 USA

**Keywords:** MAVE, Machine learning, Variant classification

## Abstract

As the adoption and scope of genetic testing continue to expand, interpreting the clinical significance of DNA sequence variants at scale remains a formidable challenge, with a high proportion classified as variants of uncertain significance (VUSs). Genetic testing laboratories have historically relied, in part, on functional data from academic literature to support variant classification. High-throughput functional assays or multiplex assays of variant effect (MAVEs), designed to assess the effects of DNA variants on protein stability and function, represent an important and increasingly available source of evidence for variant classification, but their potential is just beginning to be realized in clinical lab settings. Here, we describe a framework for generating, validating and incorporating data from MAVEs into a semi-quantitative variant classification method applied to clinical genetic testing. Using single-cell gene expression measurements, cellular evidence models were built to assess the effects of DNA variation in 44 genes of clinical interest. This framework was also applied to models for an additional 22 genes with previously published MAVE datasets. In total, modeling data was incorporated from 24 genes into our variant classification method. These data contributed evidence for classifying 4043 observed variants in over 57,000 individuals. Genetic testing laboratories are uniquely positioned to generate, analyze, validate, and incorporate evidence from high-throughput functional data and ultimately enable the use of these data to provide definitive clinical variant classifications for more patients.

**Supplementary Information:**

The online version contains supplementary material available at 10.1007/s00439-024-02691-0.

## Introduction

Variants of uncertain significance (VUSs) are frequently reported in clinical genetic testing (Burke et al. 2022; Fowler and Rehm 2024) resulting from insufficient evidence to classify the variants unambiguously as either disease-causing or benign. Data from experimental studies designed to characterize the impact of DNA variants on protein stability and function can provide strong evidence to support benign or pathogenic classifications, based on the American College of Medical Genetics and Genomics/Association for Molecular Pathology (ACMG/AMP) guidelines (Richards et al. 2015). Such functional evidence was relatively scarce in the scientific literature because effects on protein function have been experimentally characterized for only a small fraction of genetic variants. Over the past decade, high-throughput sequencing-based cellular assays, collectively termed multiplex assays of variant effect (MAVEs), have been developed to systematically characterize a wide array of molecular functions, including protein-protein interactions (Araya et al. 2012), enzymatic activity (Romero et al. 2015), regulatory potential (Kwasnieski et al. 2012), and protein stability (Matreyek et al. 2018). More recently, variant function has been assessed by comparing single cell gene expression profiles from cells expressing clinically relevant variation for a gene (Ursu et al. 2022). Unlike previous approaches, MAVEs enable the characterization of many DNA variants within a single pooled experiment.

Although MAVEs present a powerful opportunity to incorporate new, highly informative data into variant classification during clinical germline genetic testing, the use of MAVEs in clinical variant classification has been underutilized. Many MAVE experiments have been conducted for basic research rather than for clinical applications; thus, only a few dozen of the many hundreds of MAVE experiments conducted in recent years focused on genes associated with monogenic diseases. In conjunction, few studies adequately characterized the relevant molecular functions (e.g., signaling, protein stability, cell death) specifically associated with those diseases. Furthermore, incorporation of MAVE data into variant classification frameworks, especially in large clinical laboratory settings, has been hindered by a lack of detailed guidance from professional laboratory groups to independently build and evaluate the clinical quality of models derived from MAVE data.

To standardize and more efficiently use MAVE data, we employed a machine-learning-based evidence modeling platform to control the quality of models built from MAVE datasets and to incorporate those models that passed rigorous validation into routine clinical variant classification. MAVE experiments were designed and performed for 44 disease-related genes to generate new functional data for variants of clinical interest observed through genetic testing. Of these 44 models, 19 were selected to be included in variant classification. In addition, cellular models were assessed from an additional 22 genes built from MAVE datasets that were previously published by the academic community. Five of the 22 genes passed our quality control thresholds. In total, 24 cellular models (19 internal and 5 external) were integrated into clinical variant classification, providing additional evidence to classify over 4,000 variants in over 57,000 individuals.

## Materials and methods

### Cellular evidence platform for internally-generated MAVEs

For single-cell RNA-seq models developed in our laboratory, variants of interest for a target gene were selected based on whether they could be used in training models or were VUS discovered during testing by Invitae. VUS capable of being reclassified with high-quality functional evidence as well as those observed in multiple individuals were prioritized during the selection process. The selected variants were synthesized (Twist Biosciences) and designed to include unique barcodes in the 3-prime untranslated (UTR) region of the transcript. The barcoded variant pool was then cloned into a donor plasmid backbone using the DNA HiFi Assembly Mix following the manufacturer’s instructions (New England Biolabs). To identify synthesis and cloning errors and ensure high-quality starting libraries, we performed long-read sequencing of the cloned variant libraries using a Sequel IIe system (Pacific Biosciences). To introduce the variants precisely into cells, a site-specific recombination-based approach (Flp-In™ T-REx™, Thermo Scientific) was employed. First, Flp-In™ T-REx™ 293 landing pad cells were co-transfected (FuGENE6, Promega) with the variant library donor plasmid as well as a second plasmid to drive expression of the recombinase according to the manufacturer’s instructions. Forty-eight hours post-transfection, cells were split and selected with hygromycin [150 𝛍g/mL] for 9 days to isolate cells that had undergone successful recombination.

For loss-of-function targets, small interfering RNA pools (custom duplex RNAs, Horizon Biosciences) targeting the endogenous 3-prime UTR, which is not present in the exogenous copy, were transfected 72 hours prior to collecting scRNA-seq measurements, following manufacturer’s instructions (Lipofectamine™ RNAiMAX, Invitrogen). For both loss-of-function and gain-of-function targets, cell pools were exposed to doxycycline [1 𝛍g/mL] for 48 hours to induce expression of the variant library. Following induction, single-cell RNA-seq was performed following the manufacturer’s instructions (Chromium Next GEM Single Cell 3’ Kit, 10X Genomics), targeting 100 cells per variant.

Variants were associated with cells by generating a separate, targeted variant-cell barcode (VCB) library. The VCB library is derived from the single cell cDNA and contains both the variant barcode as well as the cell barcode that was introduced during the first-strand synthesis step of the scRNA-seq protocol. VCB libraries were constructed by three consecutive rounds of PCR with intervening bead (Axygen) cleanups. The first round of PCR began with 5ng of the single-cell cDNA pool and consisted of 12 cycles of PCR using a target-specific forward primer and a common reverse primer (SI-Primer, 10X Genomics) that anneals to the adapter downstream of the cell barcode. Following cleanup with 0.8x beads, a second round of 8 cycles of PCR was performed with a second internal target-specific primer and a common reverse SI-Primer and the products were purified with a dual-sided cleanup with 0.6x and 0.8x beads, respectively. A final round of PCR utilized 1ng DNA taken from the second PCR and included 8 cycles of amplification with forward and reverse primers that incorporated Illumina adapters and indices. The final amplification reaction was subjected to a dual-sided bead cleanup with 0.6x and 0.8x beads, respectively.

The VCB and RNA-seq libraries were mixed at a ratio of 30:70 and sequenced on an Illumina MiSeq instrument (Illumina). Data from the MiSeq run was used to both assess the quality of the RNA-seq libraries prior to deep sequencing and to determine the cell to variant associations (see analysis section). Following quality control, the scRNA-seq libraries were repooled, if necessary, to perform deep sequencing on an Illumina NovaSeq 6000 instrument with a target of 50,000-100,000 reads per cell.

### Analysis workflow

Consensus long-reads for each variant library were generated using SMRT link software (Ardui et al. 2018). Reads were aligned to the reference sequence for the target gene and variant barcodes were identified using constant sequences flanking the left and right of the barcode (Farrar 2007). To account for potential sequencing errors, each variant barcode was clustered using umi_tools.network (Smith et al. 2017). The set of reads corresponding to each variant barcode cluster was then examined to identify single nucleotide variants in the coding region of the target gene. For the variant to variant barcode relationship to pass quality control, the most common mutation had to be at least 50% of the total reads for a variant barcode cluster and match the designed SNV for that barcode. Additionally, we eliminated variant to variant barcode relationships from subsequent analyses where 20% or more of the reads were shorter than the expected length of the target gene by 50 bp or more.

To quantify gene expression and identify cell barcodes, CellRanger’s (version 3.1.0) mkfastq, count, and aggr tools were used for the transformation of bcl files into count matrices. To associate variants to cells, extracted variant barcodes (8 bp) were extracted from Read 2 of the VCB library as well as 10X cell barcodes (16 bp) and transcript unique molecular identifier (UMI) (12 bp) sequences from Read 1. The reference sets of variant barcodes and cell barcodes were defined from the variant to variant barcode analysis above and the filtered matrix output of CellRanger counts, respectively (Zheng et al. 2017). To account for sequencing errors, 1 bp discrepancies between the reference and extracted barcodes were allowed. For each cell barcode, unique transcript UMIs were extracted and clustered using the UMIClustering adjacency method from umi_tools.network. Valid cell to variant barcode associations fulfilled the following criteria: (1) the number of unique transcript UMIs for a given cell barcode was ≥ 3; (2) a single variant barcode achieved a > 0.5 fraction of the unique transcript UMIs for a given cell barcode; (3) the variant barcode with the second highest fraction of unique transcript UMIs did not exceed 0.25.

To remove cells of low quality, we used HDBSCAN (McInnes and Healy 2017) to cluster cells based on the following features: the total number of UMI per cell, the total number of UMI in mitochondrial genes per cell, the percentage of mitochondrial UMI in cells, the total number of genes with non zero values per cell and the total number of UMI in the highly expressed genes per cell. Next, the proportion of cells with < 5000 UMI for each cluster was calculated. Cells were removed if they were assigned to a cluster where the proportion of < 5000 UMI cells in the cluster was < 0.2.

After the data preprocessing was done, expression data for cells that passed QC and had variants identified was used for model training. The labels used for training included pathogenic and benign variants classified by Invitae Corp. and also a subset of ClinVar submissions (https://www.ncbi.nlm.nih.gov/clinvar/). Genes were removed that did not have expression in a minimum of 10% of cells for at least one variant. The mean expression of each gene, within the cells of each variant, was used as the input for modeling. Row sum normalization was used on the cells, features were selected using the index of dispersion as described by Zheng et al. (2017) and PCA was utilized to reduce dimensions. Sklearn (Pedregosa et al. 2011) was used to train models (e.g., random forests, logistic regression and SVM) and optimize hyperparameters through 3 repetitions of 5-fold cross-validation. The highest performing model was calibrated using sklearn’s CalibratedClassifierCV and leave-one-out-cross-validation (LOOCV) was repeated 10 times to produce the final pathogenicity scores for each variant.

### Subject data and clinical variant classification

This study used germline DNA variant data from individuals referred by clinicians for diagnostic genetic testing for hereditary disorders. Clinical variant classification was performed utilizing a validated system, Sherloc, based on the ACMG/AMP guidelines (Nykamp et al. 2017). Sherloc uses a semiquantitative point-based rubric for evaluating variant type, allele frequency, and clinical, functional and computational evidence. A likely benign classification and benign classification require 3 benign points and 5 benign points respectively, while a likely pathogenic classification and pathogenic classification require 4 pathogenic and 5 pathogenic points respectively. Clinical reports were generated following professional guidelines and included details on variant classifications and their corresponding evidence.

## Results

### Framework for evaluating MAVE data

**Fig. 1:**
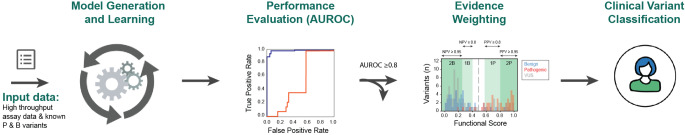
Evidence Modeling Platform. High-throughput assay data and known pathogenic (P) and benign (**B**) variant labels are used to train a model. The performance of the model is evaluated to determine whether model predictions correlate with known pathogenic and benign variants. Pathogenic (P) or benign (**B**) Sherloc points are weighted by PPV and NPV calculations, with the PPV/NPV ≥ 0.95 bin, PPV/NPV ≥ 0.8 bin and the PPV/NPV < 0.8 bin receiving 2, 1 or 0 Sherloc points, respectively. MAVE evidence is then incorporated into the larger variant interpretation system within Sherloc, which also includes evidence such as patient phenotype and family segregation

To generate, evaluate and incorporate various types of machine learning models, we developed a single Evidence Modeling Platform (manuscript in preparation). In the context of MAVE-based models, this platform uses supervised machine learning on experimental features from cellular studies to develop a gene-specific model for predicting variant pathogenicity (Fig. 1). Models that achieved high performance in discriminating between known pathogenic and benign variants (AUROC ≥ 0.8) were deemed valid. The output of these validated models, i.e., quantitative variant pathogenicity scores ranging from 0 (benign) to 1 (pathogenic), were further calibrated by calculating negative predictive value (NPV) and positive predictive value (PPV) using the known pathogenic and benign variants. Evidence weights (i.e., points in Sherloc) to variant pathogenicity scores were based on NPV and PPV thresholds. To determine the final classification, variants with this type of functional evidence were subjected to the full variant classification process by clinical genomic scientists and licensed laboratory directors.

**Fig. 2:**
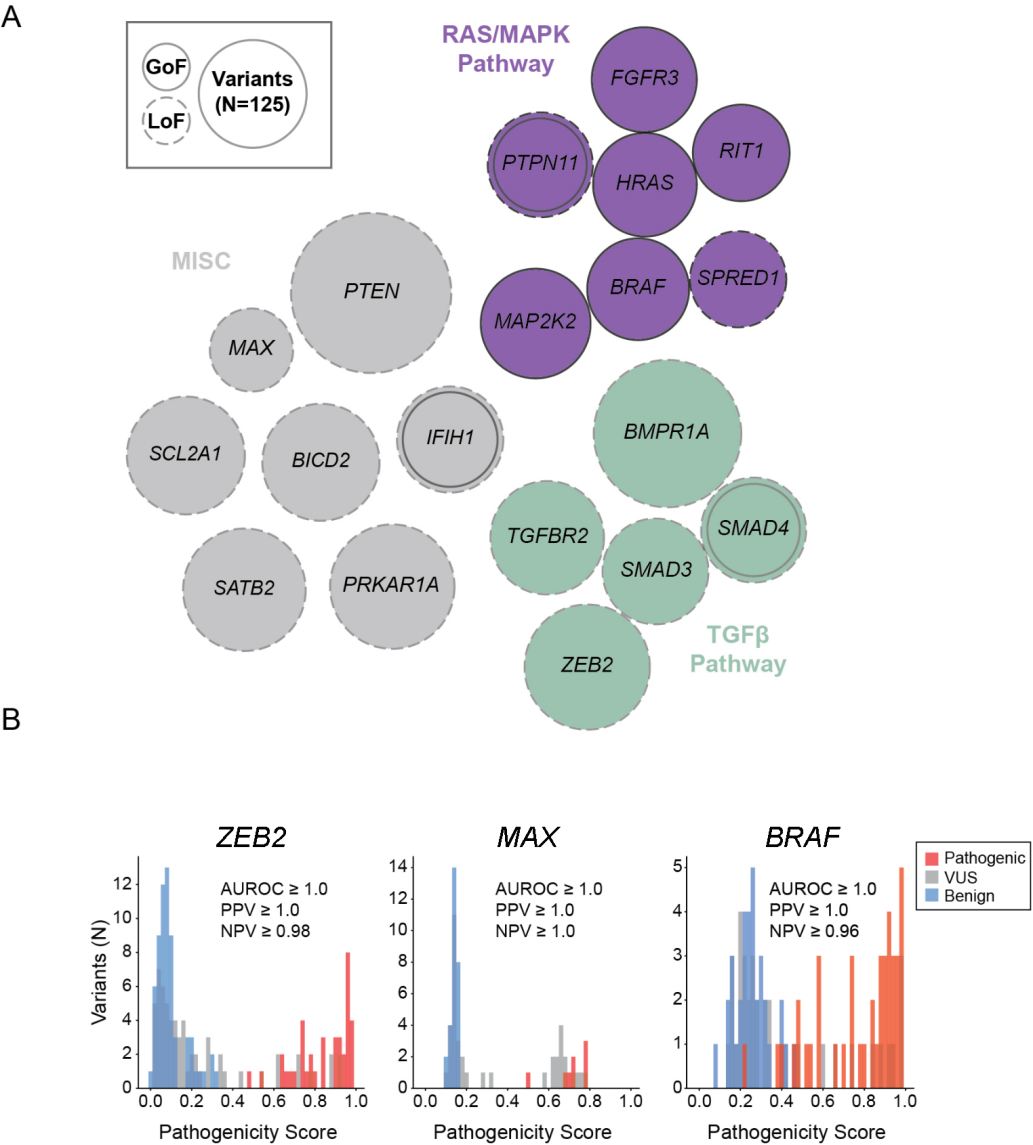
Genes with high-quality internally generated MAVE data. **A**) The size of the circles correlates with the number of variants utilized in the experiment. Of these, *MAX* was the smallest experiment with 74 variants, and *PTEN* was the largest with 274 variants. Circles that are touching represent genes that interact with each other within a biological pathway. **B**) Histograms of pathogenicity score distributions for selected datasets

**Fig. 3:**
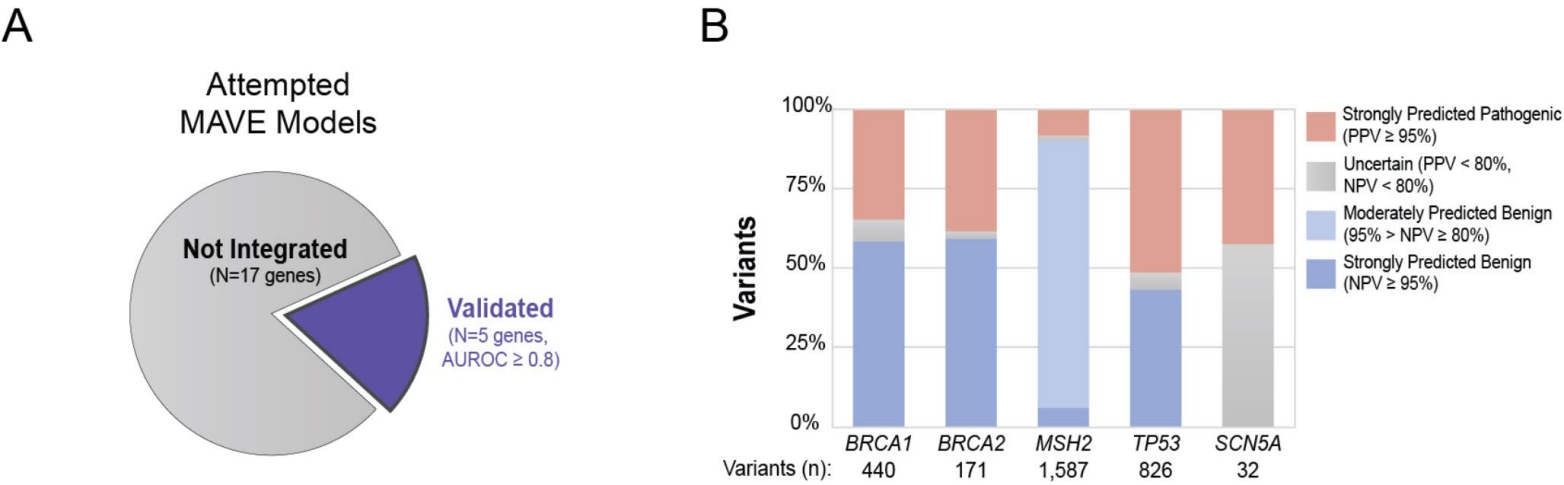
Cellular evidence models derived from published, high-throughput functional datasets. **A**) Cellular evidence modeling was attempted for published MAVE datasets for 22 genes. Models for 5 genes exhibited sufficient performance (AUROC ≥ 0.8) to be used in variant classification. **B**) Bar graph illustrating the distribution of prediction quality for the five externally derived cellular evidence models (*BRCA1*, *BRCA2*, *MSH2*, *TP53*, and *SCN5A*) that met performance thresholds. Note, the *TP53* model is derived from the combination of data from three publications (Kato 2003; Giacomelli et al. 2018; Kotler et al. 2018)

Experimental datasets from 66 genes were evaluated with our machine-learning platform. These datasets were either generated within our functional genomics laboratory (44 genes) or were obtained through publications from external groups (22 genes) (Findlay et al. 2018; Richardson et al. 2021; Jia et al. 2021; Glazer et al. 2020; Kato 2023; Giacomelli et al. 2018; Kotler et al. 2018; Weile et al. 2017; Sun et al. 2020; Majithia 2016; Mighell et al. 2018; Matreyek et al. 2018; Brenan et al. 2016; Bandaru et al. 2017; Newberry et al. 2020; Chiasson et al. 2020; Starita et al. 2013; Melamed et al. 2013; Starita et al. 2015; Raraigh et al. 2018; Araya et al. 2012; Amorosi et al. 2021). Evaluating each MAVE dataset was critical because the performance of the resulting predictive models varied widely (Supplementary Fig. 1). Of the 44 datasets produced by our laboratory using single-cell RNA sequencing, 19 yielded a predictive model that both met a performance threshold of AUROC ≥ 0.8 and were selected for integration into Sherloc (Supplementary Table 1). Unlike cell type identification via unsupervised clustering of scRNA-seq profiles, cells harboring pathogenic or benign variants are often intermixed, although analogous clustering at the variant level highlights the signals leveraged by the machine learning models to accurately classify variants (Supplemental Fig. 2). Performant models were achieved for genes across multiple biological pathways and both loss-of-function and gain-of-function disease mechanisms (Fig. 2). Of the external MAVE datasets from 22 genes evaluated, 5 predictive models (for *BRCA1*, *BRCA2*, *MSH2*, *SCN5A* and *TP53*) each met a performance threshold of AUROC ≥ 0.8 (Fig. 3, Supplementary Table 2). The majority of the remaining, unintegrated datasets either did not have enough known pathogenic and benign variants to allow assessment or showed insufficient ability to discriminate between benign and pathogenic variants (AUROC < 0.8) and were excluded from further assessments in this manuscript (Supplementary Table 3).

### Performance-based integration into a variant classification framework

To ensure that appropriate weight was accorded to the evidence generated by each performant model for the purpose of variant classification, seven tiers were devised based on the NPV and PPV of each model and to mirror existing Sherloc criteria for incorporating functional experimental data (Supplementary Table 4). The first two tiers were defined as [highly predictive benign] and [moderately predictive benign] and were awarded 2 and 1 benign points, respectively. The next two tiers were [moderately predictive pathogenic] and [highly predictive pathogenic] and were awarded 1 and 2 pathogenic points, respectively. The predictive performance thresholds for these tiers were respectively defined as (1) [highly predictive benign] > 95% NPV, (2) [moderately predictive benign] ≥ 80–95% NPV, (3) [moderately predictive pathogenic] ≥ 80–95% PPV, (4) [highly predictive pathogenic] > 95% PPV. The fifth tier corresponded to predictions below 80% PPV and below 80% NPV, which were deemed insufficiently certain to be assigned a weight within the Sherloc scoring system. As the *TP53* model was both highly predictive and developed from multiple distinct functional readouts (Kato 2003; Giacomelli et al. 2018; Kotler et al. 2018), it was awarded the final two tiers, [very highly predictive benign, > 97.5% NPV] and [very highly predictive pathogenic, > 97.5%], worth 2.5 benign points and 2.5 pathogenic points, respectively.

### Variant reclassification and impact

Within 24 genes for which available datasets yielded a performant predictive model, there were 4043 observed variants with sufficiently confident predictions (≥ 80% NPV or ≥ 80%PPV) to potentially impact Sherloc scoring (Table 1, Supplementary Table 5). To understand the impact of adding this evidence, we reevaluated a subset of variants classified as VUS (*n* = 3474) for which Sherloc points were applicable as a result of a MAVE-based model. Across genes, we observed an average VUS reclassification rate of 12.6% (436/3474) (Table 2), which included 127 unique variant upgrades from VUS to likely pathogenic/pathogenic and 309 downgrades from VUS to likely benign/benign. MAVE models also contributed to upgrading classifications for 44 variants from likely pathogenic to pathogenic, and downgrading 43 from likely benign to benign. As of Q1 2024, approximately 57,096 patient clinical reports contain variants that have evidence from these cellular models. Within these impacted reports, 38,614 have variants receiving benign evidence and 19,417 have variants receiving pathogenic evidence, with a small number of reports containing multiple variants receiving evidence.

**Table 1:** Number of unique variants per gene and corresponding Sherloc weighted scores matching predictive performance tiers

NPV/PPV	NPV80	NPV95	NPV97.5	PPV80	PPV95	PPV97.5	Total	Ref.
Sherloc score	1B	2B	2.5B	1P	2P	2.5P		
Gene								
*BICD2*	0	76	0	0	0	0	76	This study
*BMPR1A*	0	133	0	0	13	0	146	This study
*BRAF*	0	32	0	0	27	0	59	This study
*BRCA1*	0	257	0	0	153	0	410	Findlay et al. 2018
*BRCA2*	0	102	0	0	65	0	167	Richardson et al. 2021
*FGFR3*	0	0	0	0	15	0	15	This study
*HRAS*	0	36	0	0	9	0	45	This study
*IFIH1*	8	35	0	0	3	0	46	This study
*MAP2K2*	0	76	0	0	6	0	82	This study
*MAX*	0	25	0	0	0	0	25	This study
*MSH2*	1337	94	0	0	132	0	1563	Jia et al. 2021
*PRKAR1A*	0	0	0	0	9	0	9	This study
*PTEN*	21	35	0	0	81	0	137	This study
*PTPN11*	0	0	0	0	38	0	38	This study
*RIT1*	0	28	0	0	11	0	39	This study
*SATB2*	0	87	0	0	2	0	89	This study
*SCN5A*	0	0	0	0	13	0	13	Glazer et al. 2020
*SLC2A1*	31	15	0	0	8	0	54	This study
*SMAD3*	0	22	0	0	45	0	67	This study
*SMAD4*	0	0	0	4	0	0	4	This study
*SPRED1*	0	42	0	0	4	0	46	This study
*TGFBR2*	0	10	0	11	32	0	53	This study
*TP53*	0	0	356	0	0	425	781	Kato 2003, Giacomelli et al. 2018, Kotler et al. 2018
*ZEB2*	0	77	0	0	2	0	79	This study
Total	1397	1182	356	15	668	425	4043	

*Abbreviations* are as follows: NPV80, negative predictive value ≥ 80–95%; NPV95, negative predictive value > 95%; NPV97.5, negative predictive value > 97.5%; PPV80, positive predictive value ≥ 80–95%; PPV95, positive predictive value > 95%; PPV97.5, positive predictive value > 97.5%. Sherloc scores are the numerical benign (B) or pathogenic (P) point value assigned based on NPV/PPV

**Table 2:** Reclassification of variants with the incorporation of evidence from MAVE datasets

Gene	VUS > B	VUS > LB	VUS > LP	VUS > *P*	VUS > VUS	LP > *P*	LB > B	Ref.
*BICD2*	6	35	1	0	18	0	7	This study
*BMPR1A*	0	1	1	0	139	0	0	This study
*BRAF*	0	1	3	0	31	5	2	This study
*BRCA1*	0	2	13	0	297	2	3	Findlay et al. 2018
*BRCA2*	0	0	11	0	99	5	8	Richardson et al. 2021
*FGFR3*	0	0	0	0	1	0	0	This study
*HRAS*	0	0	1	0	47	2	0	This study
*IFIH1*	1	18	0	0	18	0	3	This study
*MAP2K2*	0	2	0	0	68	0	1	This study
*MAX*	0	1	0	0	26	0	0	This study
*MSH2*	75	134	5	2	1238	4	1	Jia et al. 2021
*PRKAR1A*	0	0	0	0	8	1	0	This study
*PTEN*	0	0	11	1	93	12	0	This study
*PTPN11*	0	0	1	0	6	0	0	This study
*RIT1*	0	0	0	0	33	0	0	This study
*SATB2*	12	1	1	0	46	0	12	This study
*SCN5A*	0	0	4	1	13	3	0	Glazer et al. 2020
*SLC2A1*	0	3	1	1	60	0	0	This study
*SMAD3*	0	3	5	1	51	3	0	This study
*SMAD4*	0	0	0	0	0	0	0	This study
*SPRED1*	0	1	0	0	44	0	1	This study
*TGFBR2*	0	2	2	5	35	3	1	This study
*TP53*	0	1	16	40	626	3	2	Kato et al. 2003, Giacomelli et al. 2018, Kotler et al. 2018
*ZEB2*	4	6	0	0	41	1	2	This study
Total	98	211	76	51	3038	44	43	

*Abbreviations* are as follows: B, benign; LB, likely benign; LP, likely pathogenic; P, pathogenic; VUS, variant of uncertain significance

## Discussion

Historically, genetic testing laboratories have obtained functional experimental data from the scientific literature as one of many types of evidence that contribute to variant classification. In addition to being highly distributed, the evaluation of these low-throughput assays was generally not well-standardized, qualitative in nature and prone to subjectivity, exacerbating the challenges of leveraging experimental data for variant classification. The advent of MAVE technologies presents new opportunities for genetic testing laboratories to both systematically evaluate and incorporate functional evidence into variant classification as well as to potentially generate their own experimental data. Genetic testing laboratories have large amounts of clinical data and expertise that can inform experimental design and enable targeting of genes and variants that are most likely to benefit from new functional data. To date, however, the translation of these MAVE experiments into large-scale variant classification frameworks, and therefore their impact on patients, has been limited.

This challenge is well-suited for a machine learning platform that can build evidence models with MAVE data as features and known pathogenic and benign variants as training labels. Using this approach, we analyzed MAVE datasets for 66 genes and observed that models for 24 genes could be generated that met the quality thresholds to be utilized for clinical variant classification. Many factors likely contributed to the observed performance of each model. For example, many MAVEs have characterized a single region of the gene or one aspect of a gene’s function, whereas pathogenicity is often associated with multiple molecular functions. Additionally, many published datasets included very few clinically evaluated variants in the assay with which to gauge performance. Our own experiences generating MAVE data indicate that gene expression is not a universally appropriate readout and the choice of cell type is also an important consideration. Moreover, the experiments described here were conducted in normal growth conditions and without cofactors, which may be insufficient to reveal pathogenic phenotypes. Whatever the underlying cause, the number of models that did not meet our quality thresholds caution against naively using this evidence in clinical settings and highlight the importance of a rigorous process for quality control.

Combining multiple MAVE datasets using machine learning also has the potential to enhance performance when compared to models derived from individual datasets, as this strategy mitigates the limitation highlighted above that a single MAVE dataset may only measure one aspect of gene function. Therefore, a single model trained with multiple overlapping datasets may result in improved positive and/or negative predictive values, thereby making it better suited for variant classification. Indeed, this is what we and others (Fayer et al. 2021) have observed for *TP53*. *PTEN* represents another opportunity for such combined modeling, as multiple functional datasets exist and additional benign variants have been classified in recent years. Although existing guidelines (Fortuno et al. 2021) for incorporating *TP53* MAVE datasets into clinical variant classification also attempt to address these limitations, they are challenging to implement at scale and difficult to envision as a robust strategy for handling the increasing volume of MAVE datasets being released. For example, the guidelines are only applicable to *TP53*, so cannot be specifically utilized for other genes, and any new *TP53* MAVE datasets cannot be readily integrated into the existing workflow. As new MAVE datasets will continue to be generated both internally and externally, machine learning allows for a more streamlined and efficient integration of performant models into clinical variant classification, decreasing the time from data generation to clinical impact. Moreover, models can be continuously evaluated, compared and updated using a level playing field and not by subjective assessment.

One distinct aspect of the approach described here is the method of assigning an evidence strength to the prediction scores provided by the MAVE models. In this approach, we determined predictive value bins for each variant score, and the evidence weight assigned to a prediction within the Sherloc classification framework scaled with the predictive value. Consistent with joint ACMG/AMP guidelines, we set the maximum weight of evidence from the MAVE models at 2.5 points, or the equivalent of Strong Evidence described in the ACMG/AMP framework. As a result, additional non-functional evidence is required to reach a definitive likely benign or likely pathogenic classification. In 2019, the ClinGen Sequence Variant Interpretation Working Group further recommended utilizing odds of pathogenicity (OddsPath) as a metric for assigning evidence strength to functional datasets (Brnich et al. 2020). Both our strategy and OddsPath are important quality control measures and seek to assess the performance of the evidence and to scale weighting in variant classification frameworks by the performance. In contrast to the OddsPath recommendations, our approach explicitly allows for multiple evidence weight bins for a given MAVE model. Although reclassification rates are influenced by a number of factors (see below), we have generally observed lower rates upon integrating models built from common datasets. Nevertheless, we have used this strategy for all model types within the Evidence Modeling Platform to significantly reduce VUS rates across a wide diversity of genes without impacting the quality of the classifications (Chen et al. 2023).

With our integration methods, MAVE models enabled reclassification of 12.6% (436/3474) of VUSs observed by our laboratory. Other studies (Fayer et al. 2021; Kim et al. 2020; Scott et al. 2022) have demonstrated a wide range of reclassification rates (0.11 − 74%) owing to the use of MAVE-derived evidence. A point to consider when reviewing reclassification rates is that the baseline amount of evidence that a clinical lab has on a variant will impact the rate of VUS reclassification. This can vary from laboratory to laboratory based on a number of different factors, including the number of patient samples tested as well as the sources of functional evidence. The reclassification rate at any given point in time is also an incomplete measure of the impact of MAVE models. For the many VUS in which reclassification has not yet been achieved, the application of this evidence will lessen the amount of additional evidence required for them to reach a terminal classification and thereby reduce the time they would otherwise spend as a VUS.

As more healthcare providers utilize genetic testing for diagnostic confirmation and treatment decisions, the continued development of scalable and innovative approaches for resolving VUSs is critical. Although MAVEs represent such an approach and have been commonly used for academic research purposes, they have not yet reached their full potential in diagnostic testing settings directly. When used with proper safeguards and validation procedures, the benefits of broadly utilizing this class of evidence in clinical variant classification are clear. Indeed, approximately 57,096 patients have a report with variants impacted by the 24 MAVE models integrated here. Importantly, however, the value of MAVE data extends far beyond providing additional evidence for germline variant classification. Insights derived from MAVE experiments can also contribute to a deeper understanding of disease mechanisms as well as help to guide somatic variant classification, selection of therapeutic interventions and drug development.

### Electronic supplementary material

Below is the link to the electronic supplementary material.

Supplementary Material 1

Supplementary Material 2

## Data Availability

Code to reproduce the analysis has not been deposited in a public repository because it is proprietary, but can be made available from the corresponding author on request. Additionally, all variants have been shared with ClinVar in a de-identified manner: https://www.ncbi.nlm.nih.gov/clinvar/submitters/500031/.
