# Supplementary material for: Scalable approaches for generating, validating and incorporating data from high-throughput functional assays to improve clinical variant classification
Source: Hum Genet. 2024 Aug 1;143(8):995–1004. doi: 10.1007/s00439-024-02691-0 (PMC11303574; doi:10.1007/s00439-024-02691-0)
Supplement: Supplementary file 1 — Supplementary Material 1 [file 439_2024_2691_MOESM1_ESM.docx]

**SUPPLEMENTARY FIGURES & TABLES**


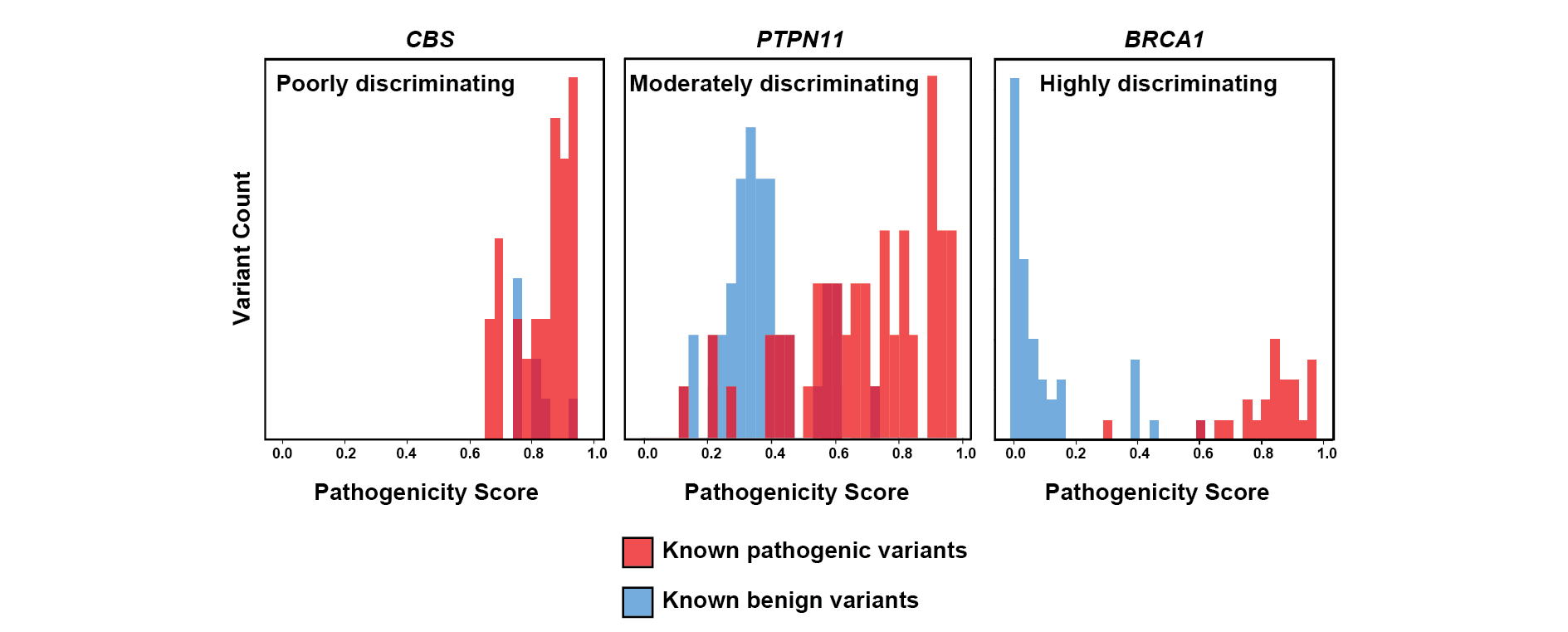


**Supplementary Figure 1. Evaluation of MAVE datasets using the evidence modeling platform**. Histograms for *CBS* (Sun 2020), *PTPN11* (Internal), and *BRCA1* (Findlay 2018) show predicted pathogenicity based on the assay data (x-axis) for known pathogenic and benign variants.


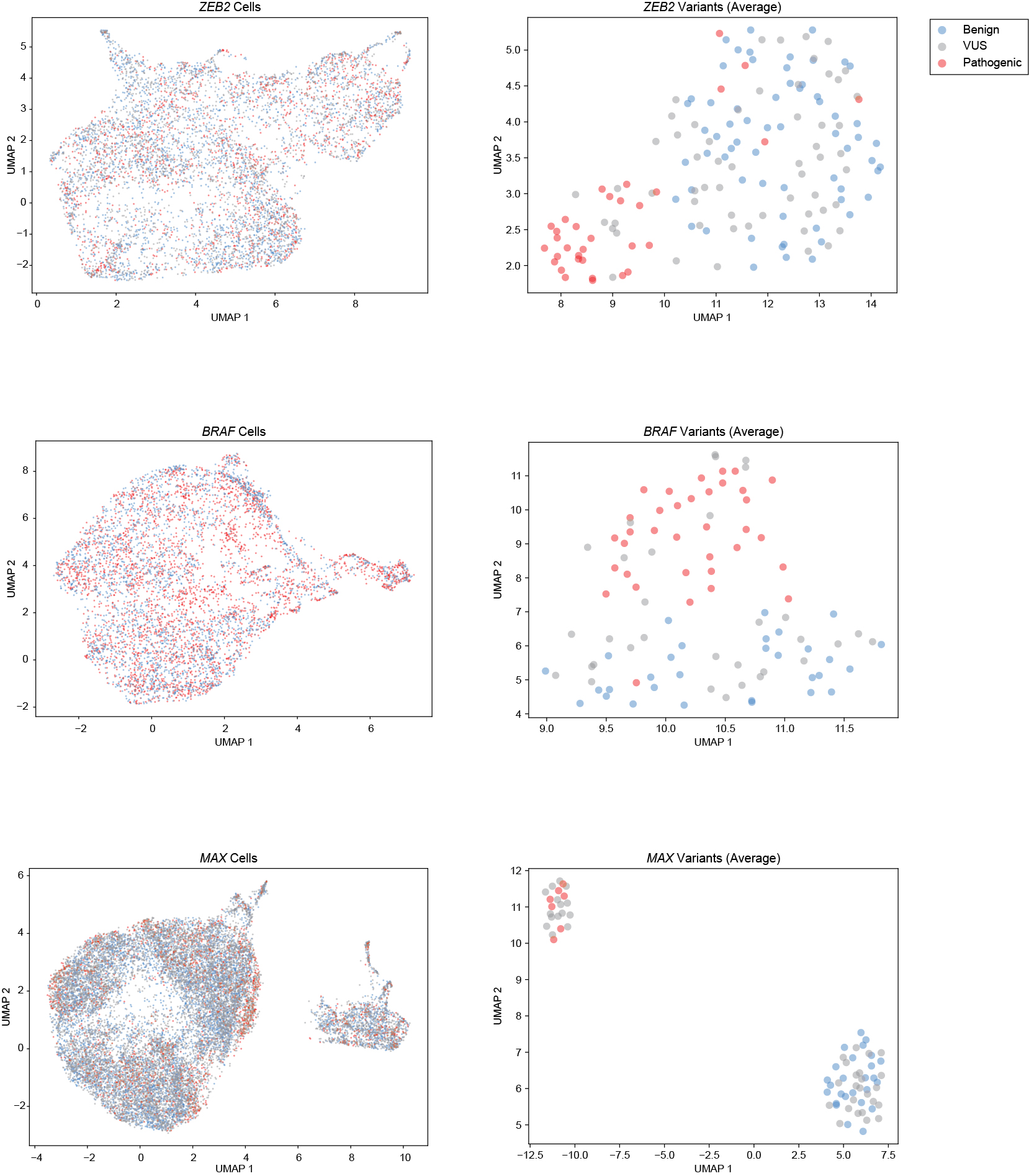


**Supplementary Figure 2: Unsupervised clustering of cells and variants.** Unsupervised UMAP (Uniform Manifold Approximation and Projection) clustering of scRNA-seq data at the cell level (left) and variant level (right) for three example genes.

**Supplementary Table 1**: List of 19 genes with internally generated cellular evidence datasets that passed quality control and were integrated into clinical variant interpretation

| Gene | AUROC | Benign Labels | Pathogenic Labels | Total number of variants | Total number of cells |
| --- | --- | --- | --- | --- | --- |
| *BICD2* | 0.89 | 54 | 12 | 144 | 16863 |
| *BMPR1A* | 0.99 | 53 | 39 | 233 | 12811 |
| *BRAF* | 0.99 | 33 | 43 | 111 | 9574 |
| *FGFR3* | 0.99 | 50 | 21 | 122 | 13718 |
| *HRAS* | 0.91 | 33 | 16 | 114 | 10014 |
| *IFIH1* | 0.81 | 32 | 9 | 107 | 8431 |
| *MAP2K2* | 1 | 43 | 12 | 129 | 13160 |
| *MAX* | 1 | 27 | 8 | 74 | 21103 |
| *PRKAR1A* | 0.97 | 37 | 15 | 165 | 15567 |
| *PTEN* | 0.96 | 76 | 104 | 274 | 16569 |
| *PTPN11* | 0.88 | 41 | 55 | 118 | 12111 |
| *RIT1* | 0.98 | 28 | 17 | 100 | 13743 |
| *SATB2* | 0.99 | 59 | 28 | 161 | 10043 |
| *SLC2A1* | 0.8 | 51 | 37 | 149 | 18212 |
| *SMAD3* | 0.98 | 27 | 27 | 120 | 15567 |
| *SMAD4* | 0.91 | 46 | 29 | 118 | 14547 |
| *SPRED1* | 1 | 41 | 15 | 99 | 12797 |
| *TGFBR2* | 0.94 | 37 | 44 | 135 | 6074 |
| *ZEB2* | 1 | 64 | 38 | 163 | 7037 |

**Supplementary Table 2**: List of 5 genes from externally published datasets that passed quality control and were integrated into clinical variant interpretation

| Gene | AUROC | Benign Labels | Pathogenic Labels | Total number of variants^a^ | Reference |
| --- | --- | --- | --- | --- | --- |
| *BRCA1* | 0.98 | 57 | 101 | 1695 | Findlay 2018 |
| *BRCA2* | 1 | 115 | 44 | 246 | Richardson 2021 |
| *MSH2* | 0.91 | 90 | 69 | 5045 | Jia 2021 |
| *SCN5A* | 0.84 | 9 | 14 | 80 | Glazer 2020 |
| *TP53* | 0.99 | 73 | 200 | 2207 | Kato 2003; Giacomelli 2018; Kotler 2018 |

^a^This represents the single nucleotide variant accessible missense reported in these datasets.

**Supplementary Table 3**: List of 42 genes where modeling was attempted but not integrated into clinical variant classification in this study

| hgnc.symbol | AUROC | Benign Labels | Pathogenic Labels | Reference |
| --- | --- | --- | --- | --- |
| *THAP1^a^* | NA | 19 | 7 | This study |
| *SMARCAL1^a^* | NA | 54 | 23 | This study |
| *RAD51D^b^* | 0.55 | 48 | 28 | This study |
| *NF1^b^* | 0.47 | 71 | 48 | This study |
| *CASR^c^* | 0.95 | 55 | 49 | This study |
| *CDH1^c^* | 0.86 | 105 | 44 | This study |
| *DSG2^b^* | 0.63 | 50 | 24 | This study |
| *RAB27A^b^* | 0.10 | 21 | 9 | This study |
| *SMC1A^b^* | 0.31 | 15 | 24 | This study |
| *TP63^d^* | 0.96 | 49 | 41 | This study |
| *SMARCB1^b^* | 0.63 | 28 | 18 | This study |
| *RPE65^b^* | 0.22 | 31 | 50 | This study |
| *STK11^c^* | 0.88 | 89 | 47 | This study |
| *TTR^b^* | 0.30 | 22 | 47 | This study |
| *PIK3CA^b^* | 0.66 | 51 | 19 | This study |
| *RAF1^d^* | 0.98 | 54 | 20 | This study |
| *PIK3R1^b^* | 0.52 | 28 | 9 | This study |
| *CASK^b^* | 0.67 | 34 | 33 | This study |
| *KIF5A^b^* | 0.36 | 49 | 13 | This study |
| *ITGB2^b^* | 0.49 | 33 | 12 | This study |
| *STAT3^b^* | 0.64 | 40 | 35 | This study |
| *BMPR2^d^* | 0.95 | 64 | 22 | This study |
| *MLH1^b^* | 0.64 | 90 | 53 | This study |
| *PMS2^b^* | 0.67 | 105 | 31 | This study |
| *SOS1^d^* | 0.93 | 82 | 26 | This study |
| *CBS^b^* | 0.66 | 8 | 43 | Sun 2020 |
| *VKOR^e^* | NA | 4 | 0 | Chiasson 2020 |
| *UBE4B^e^* | NA | 0 | 0 | Starita 2013 |
| *PABPC1^e^* | NA | 0 | 0 | Melamed 2013 |
| *SNCA^e^* | NA | 1 | 1 | Newberry 2020 |
| *PTEN^e^* | NA | 4 | 111 | Matreyek 2018; Mighell 2018 |
| *TPMT^e^* | NA | 2 | 0 | Matreyek 2018 |
| *CYP2C9^e^* | NA | 5 | 0 | Amorosi 2021 |
| *PPARG^e^* | NA | 4 | 7 | Majithia 2016 |
| *HRAS^e^* | NA | 3 | 22 | Bandaru 2017 |
| *UBE2I^e^* | NA | 0 | 0 | Weile 2017 |
| *CALM1^e^* | NA | 0 | 8 | Weile 2017 |
| *SUMO1^e^* | NA | 0 | 0 | Weile 2017 |
| *TPK1^e^* | NA | 1 | 2 | Weile 2017 |
| *MAPK1^e^* | NA | 0 | 1 | Brenan 2016 |
| *CFTR^e^* | NA | 3 | 40 | Raraigh 2018 |
| *YAP1^e^* | NA | 0 | 0 | Araya 2012 |

^a^Failed scRNA-seq quality control prior to modeling; ^b^Failed model performance quality control; ^c^Flagged by clinical scientists for further evaluation; ^d^Targeted for subsequent integration; *^e^*Insufficient label number at time of training.

**Supplementary Table 4:** Cellular evidence models Sherloc points points based on negative and positive predictive values (NPV and PPV, respectively) thresholds

| Cellular evidence modeling score | Sherloc Points | Accuracy |
| --- | --- | --- |
| Very highly predictive benign score | 2.5 B | >97.5% NPV |
| Highly predictive benign score | 2 B | >95% NPV |
| Moderately predictive benign score | 1 B | ≥80% to 95% NPV |
| Moderately predictive pathogenic score | 1 P | ≥80% to 95% PPV |
| Highly predictive pathogenic score | 2 P | >95% PPV |
| Very highly predictive pathogenic score | 2.5 P | >97.5% PPV |
| Uncertain score | 0 P | < 80% NPV or < 80% PPV |

**Supplemental Table 5**: List of variants that achieved sufficiently confident predictions (≥80% NPV or ≥80%PPV) and their pathogenicity scores from 24 genes
